# Susceptibility Weighted Imaging: An Effective Auxiliary Sequence That Enhances Insight Into the Imaging of Stroke

**DOI:** 10.7759/cureus.24918

**Published:** 2022-05-11

**Authors:** Sanjay M Khaladkar, Vijetha Chanabasanavar, Satvik Dhirawani, Vaishnavi Thakker, Darshana Dilip, Vinay Kumar Parripati

**Affiliations:** 1 Department of Radiology, Dr. D. Y. Patil Medical College, Hospital and Research Centre, Pune, IND; 2 Department of Radiology, Dr. D Y Patil Medical College, Hospital and Research Centre, Pune, IND

**Keywords:** magnitude, phase, stroke, haemorrhage, infarct, susceptibility weighted imaging

## Abstract

Aim: To evaluate the utility of susceptibility-weighted imaging (SWI) sequence in stroke imaging and assess supplemental information provided by SWI in an acute stroke scenario.

Materials and methods: In this study, the appearance of cerebrovascular stroke on the SWI images were analyzed in 50 patients who presented with acute-onset neurological symptoms.

Results: Brain MRI with SWI was performed on 50 patients presenting with acute neurological symptoms. The majority were males, 32/50 (64%) and 18/50 (36%) were females. Most of the patients were in the age group > 60 years (36%), followed by 50-60 years (22%). Most of the patients had bilateral pathology, 20 (40%). The majority of patients had supratentorial lesions 34 (68%). Among 50 patients, the majority of patients had arterial stroke 20 (40%) and cerebral venous sinus thrombosis (CVST) 20 (40%) followed by amyloid angiopathy five (10%), and five (10%) had hypertensive microhemorrhage. Among the 20 patients with arterial stroke, the majority had middle cerebral artery (MCA) thrombosis 10 (50%) and among the 20 patients with venous thrombosis, eight (40%) patients had hemorrhagic infarcts. SWI was better as compared to computed tomography (CT) (P<0.05) in the detection of hemorrhagic transformation of arterial infarct, cerebral hemorrhagic venous sinus thrombosis, hemorrhagic venous infarct, hypertensive microhemorrhage, and cerebral amyloid angiopathy.

Conclusion: SWI is a useful imaging sequence that provides additional information on stroke patients. SWI requires only an additional three-four minutes to perform and can be easily incorporated into standard stroke protocol. SWI can identify various features such as hemorrhage, intraarterial thrombus, or concomitant microbleeds that are of prognostic value and affect therapeutic decisions.

## Introduction

Haacke in 1997 first proposed and patented SWI imaging, which was subsequently implemented after 2004 on commercial scanners [1]. Susceptibility is an expression of paramagnetic components post hemorrhages like ferritin and hemosiderin. The presence of these paramagnetic components results in deterioration of MR signal from that tissue, suggesting an interpreter of hemorrhagic incidence [2]. It accentuates susceptibility differences in tissues showing acute hemorrhage, acute thrombus, deoxygenated blood in veins, hemosiderin, calcium, and iron, which exhibit susceptibility differences from their surroundings. Acute stroke can show acute thrombus, deoxygenated blood in veins, and acute hemorrhage, and hence is helpful in stroke evaluation. It increases the conspicuity of hemorrhage nearly sixfold as compared to GRE. Hence, it should be included as part of routine imaging of the brain in stroke [3].

## Materials and methods

Data collection and patient selection

This was a study performed in a tertiary care center among 50 patients who presented with acute neurological symptoms such as limb weakness, loss of consciousness, and sudden onset of severe headache. Before starting this study, ethics committee approval was obtained.

Imaging acquisition

A 3-T MRI machine (Vida; Siemens) was used. Our routine brain protocol includes axial T1-weighted (T1WI), axial T2-weighted (T2WI), axial fluid attenuation inversion recovery (FLAIR), axial diffusion-weighted imaging (DWI), axial apparent diffusion coefficient (ADC), T1WI sagittal, and T2WI coronal. Magnetic resonance venography (MRV), and magnetic resonance angiography (MRA) were done if indicated. Acquisition of SWI sequence: imaging parameters were repetition time of 27 ms, time to echo 20 ms, fractional anisotropy of ten degrees, 1.0 × 0.99 mm of pixel size, 1.8 mm slice thickness, the field of view (FOV) of 200 mm2, 270 s acquisition time.

Imaging analysis

Experienced radiologists interpreted and evaluated all brain MRI images. On phase sequence of SWI hemorrhage appeared hyperintense and the calcification appeared hypointense in a left-handed system.

## Results

General findings

Brain MRI with SWI was obtained in 50 patients. Out of the 50 patients, 32 (64%) were males and 18 (36%) were females. The majority were of the age group > 60 years (36%), followed by 50-60 years (22%). The ages ranged from 28 to 76 years, and the majority of patients were from the age group > 60 years (23%), followed by 51-60 years (22%).

MRI findings

Side and location and distribution of pathology: Most of the patients had bilateral pathology 20 (40%) followed by right 14 (28%) and left side 14 (28%) and two (4%) involved brainstem. The majority of patients had supratentorial lesions 34 (68%), followed by both supratentorial and infratentorial lesions 11 (22%) and five (7%) had infratentorial lesions. Among 50 patients, the majority of patients had venous sinus thrombosis 20 (40%) and arterial stroke 20 (40%) followed by cerebral amyloid angiopathy five (10%), and five (10%) had hypertensive microbleeds (Table 1).

**Table 1:** Distribution according to brain pathology

Brain Pathology	No. of patients (n=100) *	Percentage
Arterial Stroke	20	40
Cerebral Amyloid angiopathy	5	10
Hypertensive microbleeds	5	10
Cerebral venous sinus thrombosis	20	40
Total	50	100

Arterial Stroke

In our study, among 20 patients with arterial stroke, the majority had middle cerebral artery (MCA) thrombosis 10 (50%) followed by anterior cerebral artery (ACA) thrombosis in six (30%) and posterior cerebral artery (PCA) thrombosis in three (20%) and one (6.67 %) patient had internal cerebral artery (ICA) thrombosis (Table 2).

**Table 2:** Distribution according to arterial stroke

Arterial stroke	No. of patients (n=15)	Percentage
ACA thrombosis	06	30.00
MCA thrombosis	10	50.00
PCA thrombosis	03	20.00
ICA thrombosis	01	6.67
Total	20	100

**Figure 1:**
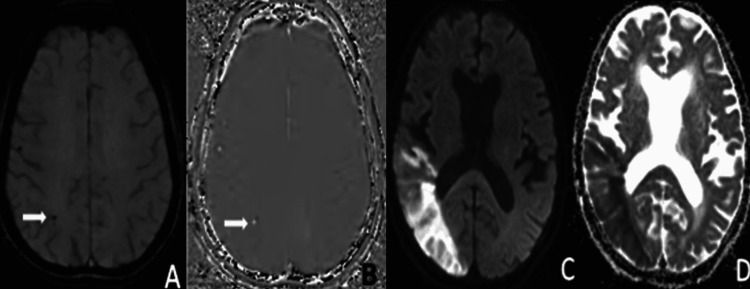
A well-defined rounded hypointense area on magnitude image (A) appearing hyperintense on phase (B) in seen in the right occipital region (white arrow) suggestive of microhaemorrhage. A large area of diffusion restriction on DWI (C) and corresponding low ADC (D) in seen in the right parieto-occipital region, suggestive of right posterior MCA territory acute infarct with microhaemorrhage within infarcted area.

**Figure 2:**
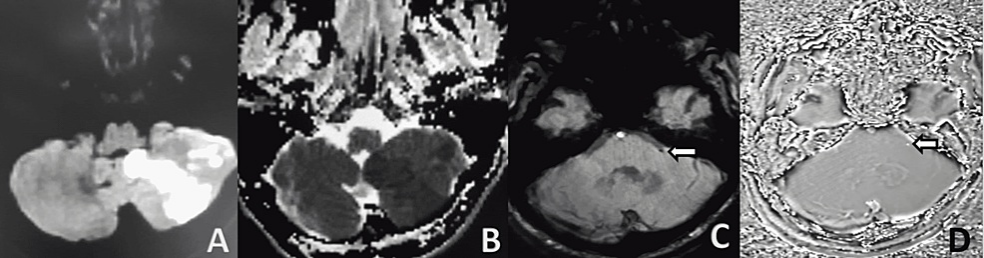
Acute non-haemorrhagic infarct noted in left cerebellar hemisphere showing diffusion restriction on DWI (A) with corresponding low ADC values (B). On magnitude left anterior inferior cerebellar artery (white arrows) appears hypointense (C) appearing bright on phase image (D) suggestive of acute thrombus.

Hemorrhagic transformation of arterial stroke was seen in 18 patients. The distribution of the hemorrhage transformation of infarcts was: MCA territory ‑ nine, ACA territory ‑ five, PCA territory ‑ five, ICA territory - one. Of the 18 patients with hemorrhagic transformation of arterial infarct, five had macrohemorrhage and 13 had microhemorrhage within the infarct area (Figures 1A-1D, 2A-2D).

**Figure 3:**
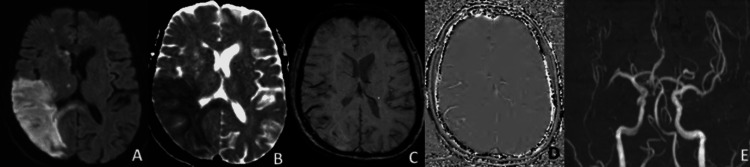
DWI (A), ADC (B), Magnitude (C), and phase (D) images of SWI sequence show infarction in left MCA territory with dark vessel sign in M3 and M4 branches of right MCA, which was confirmed by MR angiography (E).

The dark artery sign, or susceptibility sign, was seen in three patients (Figures 3A-3E).

Cerebral Venous Sinus Thrombosis (CVST)

**Figure 4:**
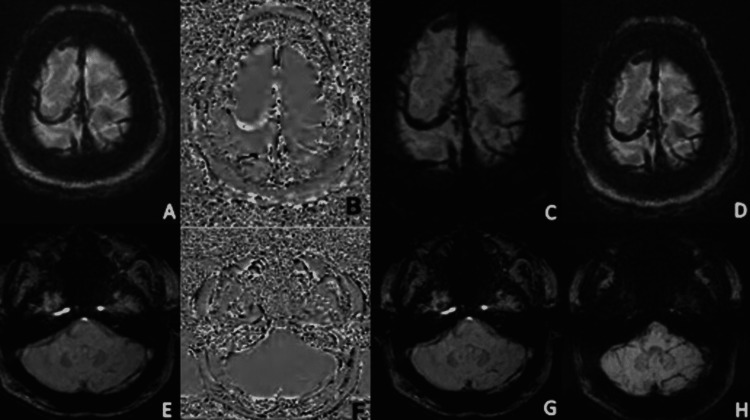
Magnitude (A.E), Phase (B,F) , SWI (C,G) and mIP (D,H) images of SWI sequence show hypointense signal in cortical veins(A,C) in bilateral fronto-parietal region and left transverse sinus (E,G) and appearing hypointense on magnitude and hyperintense on phase images (B,F) suggestive of cortical and dural venous sinus thrombosis.

Among the 20 patients with venous thrombosis, the majority of patients had hemorrhagic infarcts eight (40%) (Table 3) followed by cortical venous thrombosis in four (20%) and venous sinus thrombosis in eight (40%) cases (Figures 4A-4H).

**Table 3:** Distribution according to venous thrombosis

Venous thrombosis	No. of patients (n=10)	Percentage
Cortical venous thrombosis	04	20
Superior Sagittal Sinus thrombosis	04	20
Sigmoid sinus thrombosis	03	15
Transverse sinus thrombosis	01	5
Haemorrhagic infarct	08	40
Total	20	100

 

Correlation between CT and SWI: SWI was better as compared to CT (P<0.05) in the detection of hemorrhagic venous sinus thrombosis, hemorrhagic transformation of arterial infarct, cerebral amyloid angiopathy hypertensive, and microhemorrhage (Table 4).

**Table 4:** Correlation of CT and SWI in detection of various brain pathology

Brain Pathology	Total	Detected on SWI	Detected on CT	P value
Cerebral venous sinus thrombosis	12	12	5	0.002
Haemorrhagic venous infarct	8	8	2	0.003
Haemorrhagic transformation of arterial infarct	18	18	5	0.0001
Hypertensive Microbleeds	5	5	0	0.004
Amyloid angiopathy	5	5	0	0.004

## Discussion

SWI augments the susceptibility differences in tissues commonly associated with acute strokes, such as deoxyhemoglobin (seen in thrombosed veins that have deoxygenated blood), hemoglobin (seen in hemorrhage), and calcium [3].

SWI is a four-minute scan with four image sets. The first set is the magnitude, which shows susceptibility changes that are three to six times more visible than GRE. The phase image shows the phase of susceptibility and helps in differentiating calcification from hemorrhage. Calcium (a diamagnetic substance) has a phase that is the reverse of hemoglobin moieties and hence will appear dark, while hemorrhage will appear bright. SWI combines phase masks with magnitude images. mIP is generated by projecting the minimum signal within a voxel over several slices (usually four in number) and helps in the detection of magnetic susceptibility structure or pathology and continuity of the venous system [4].

SWI images depict slow-flowing blood in small cerebral vessels due to the blood oxygen level-dependent (BOLD) effect of deoxygenated hemoglobin, which is difficult to detect at the time of flight and phase-contrast magnetic resonance angiography techniques. mIP helps in establishing continuity of tortuous structures and has a venogram effect, thus differentiating veins from adjacent hemorrhage. Both calcium and iron are commonly deposited substances in the brain. These distort the local magnetic field, producing a low signal on the SWI image, thus mimicking hemorrhage. Calcium can be differentiated from a hemorrhage on a phase image where calcium appears dark and hemorrhage appears bright. Iron is usually seen in the globus pallidus and substantia nigra [4].

Acute thromboembolus has high iron content and an increase in deoxyhemoglobin content and hence is easily picked up on the SWI sequence. Even peripheral smaller thromboembolus or fragmented thrombi distal to an occlusive thromboembolus can be picked up on SWI, which are usually not seen on time of flight magnetic resonance angiography (TOF MRA). SWI has high spatial resolution and is a three-dimensional gradient echo sequence. It has both magnitude and raw phase data. The magnitude of the MRI signal depends on net magnetization within a voxel. For a left-handed system, diamagnetic substances (calcification) weaken the external magnetic field with a resultant negative phase shift, while paramagnetic substances (all stages of hemoglobin degradation except oxyhemoglobin) strengthen the external magnetic field, producing a positive phase shift. Hence, calcification has low signal intensity while hemorrhage has high signal intensity on phase images. Comparison is done with the signal intensity of normal venous structures like the superior sagittal sinus and superficial cortical veins. A paramagnetic substance (hemorrhage) demonstrates the same signal shift as in normal veins [5].

SWI has high sensitivity with better contrast resolution in the detection of thromboembolus in both the anterior and posterior circulation. The composition of the thromboembolus can vary. A red thromboembolus (erythrocyte rich) predominantly consists of erythrocytes, while a white thromboembolus is composed of platelets, fibrin, and atheromatous gruel (cholesterol, foam cells, and fibrous caps). A red thrombus usually arises from the left atrium in atrial fibrillation, while a white thrombus (fibrin/platelet rich thromboembolus) originates from vulnerable atherosclerotic plaques [6]. Red thrombus usually has a higher level of paramagnetic content (deoxyhemoglobin, methaemoglobin, and oxidised ferric iron). It causes blooming on the SWI sequence with the diameter of the thrombosed vessel exceeding the diameter of the contralateral normal vessel [7].Detection of red thrombus is of prognostic importance as it is more sensitive to intravenous tissue plasminogen activator treatment (IV-tPA) with a better success rate of endovascular recanalization [8].

The length and extent of thromboembolus in acute ischemic stroke are useful in the success of reperfusion treatment. Proximal thromboembolus location and length greater than 15 mm are poor predictors of IV-tPA reperfusion [9,10]. SWI is more effective in locating the distal end of thromboembolus [11]. It depicts native vessel morphology as the thromboembolus naturally outlines the vessel and hence can estimate vessel angulation. Higher angulation between ICA and the M1 segment of MCA, as well as the M1 and M2 segments of MCA, are associated with a lower rate of recanalization. Curved vessel walls increase wall friction and cause an infolding effect at the proximal and distal vessel, thus preventing the passage of stent retrieval devices [12].

SWI is extremely useful in the evaluation of vertebrobasilar (posterior) circulation. Dissection of the vertebrobasilar artery can cause distal embolization and cerebellar infarcts in young adults. Both intramural hematoma and atheroma calcification appear hypointense on SWI. Intramural hematoma is crescentric in morphology, shows positive phase shift, and hence appears hyperintense on phase images. Atheroma calcifications are concentric, eccentric, and linear and appear hypointense on phase images as they cause negative phase shift [13].

Fragmented thrombi occur due to fragmentation of primary thromboembolus or the presence of one or more thromboembolus. Its identification is important for prognostic purposes as it is a predictor of larger infarct volume, reduced collateral circulation, increased stroke severity and hence higher disability. SWI can pick up fragmented thrombi and their location, thus providing information for neuro-intervention [14].

Calcified thromboembolus arises from carotid atherosclerotic plaque, aortic arch atherosclerotic plaque, calcified aortic stenosis, and mitral annular calcification. It can occur after interventional or cardiothoracic procedures [15]. Its detection is of prognostic importance as t-PA is ineffective with high chances of recurrent ischemia. SWI can effectively detect calcified thromboembolus [16].

SWI can detect distally located clots that may not be picked up on routine MRA. Thrombus in a partially occluded vessel cannot be picked up in TOF MRA, which shows a narrowed artery. SWI can pick up thrombus at the site of an occluded vessel, seen as the dark vessel sign. SWI can pick up venous changes at the site of the infarct. Multiple prominent hypointense veins can be seen in the vicinity of the infarct due to increased oxygen extraction with a resultant increased concentration of deoxyhemoglobin. This helps in predicting tissue at risk in the penumbra and has a favorable outcome from the reperfusion strategy [17].

Acute infarction with or without hemorrhage results from cerebral-vascular ischemia caused by thromboembolism or arteriosclerotic stenosis. Because hemorrhagic transformation following a stroke can be life-threatening and is a contraindication to the use of anticoagulant and thrombolytic medicines, accurate early diagnosis of hemorrhage is critical. The National Institute of Neurological Disorders and Stroke investigations have identified three kinds of parenchymal hemorrhages, i.e., hemorrhagic infarcts (HI), parenchymal hematomas (PH), and extra-ischemic hematomas. HI indicates petechial hemorrhages that are categorized as HI-1 for isolated petechial hemorrhages or HI-2 for confluent petechial hemorrhages. PH refers to parenchymal hematoma, PH1 for hematoma that occupies < 30% of the infarct zone and PH2 for hematoma that occupies > 30% of the infarct zone and has a large mass effect. According to research, HI1, HI2, and PH1 are associated with a lower risk of premature cognitive impairment and death than PH2. SWI has greater sensitivity over T2* GRE for the detection of microhemorrhages and allows identification of HI1 and H12 patterns [18].

In our study, among 20 patients with arterial stroke, the majority of patients had MCA thrombosis, followed by ACA and PCA thrombosis. Our findings were in favor of Abdelgawad et al. [19], where SWI detected intra-arterial thrombus in 122 patients compared to 97 patients detected by MRA.

The intra-arterial thrombus is characterized by considerable hypointensity in the arteries with an apparent increase in vessel diameter compared to the contra-lateral vessels due to a “blooming” artefact, also called the susceptibility sign [20]. This is similar to the hyper-dense MCA seen on CT scans [7]. The dark artery sign, or susceptibility sign, was seen in three patients.

All hemoglobin forms except oxyhemoglobin are paramagnetic with unpaired electrons and cause susceptibility. The presence or absence of intraparenchymal hemorrhage is an important determinant of thrombolytic treatment and of prognostic importance. It can occur at the time of acute infarct or post therapy after reperfusion and can occur as a complication of thrombolysis. Its detection is important when stroke therapy is initiated three hours after the onset of hemorrhage. SWI can identify very minute hemorrhages within the infarct because it is extremely sensitive to magnetic field inhomogeneity [21]. Microangiopathies present as multiple cerebral microbleeds, most frequently seen in hypertensive and amyloid angiopathies. Cortical-subcortical region bleeds are indicative of cerebral amyloid angiopathy, and microbleeds involving the thalamus and basal ganglia are due to hypertension [22]. Chronic hemorrhages in a stroke patient may represent the fragile vascular system and have been recommended by some authors as a predictor of future bleeds, particularly in patients receiving thrombolytic therapy [5,23,24]. These microbleeds have been identified as a possible predictor of ICH risk in stroke patients [25,26]. High cerebral microbleed burden (more than 10) in patients with acute stroke treated with IV tPA is associated with a higher risk of symptomatic intracerebral hemorrhage [27].

**Figure 5:**
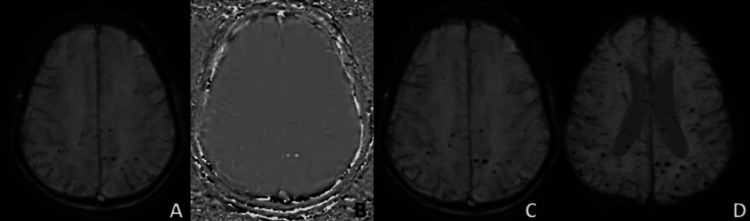
Magnitude (A), Phase (B), SWI (C), and mIP (D) images of the SWI sequence show hypointense areas on magnitude images, hyperintense on phase in the bilateral temporo-occipital region suggestive of amyloid angiopathy.

**Figure 6:**
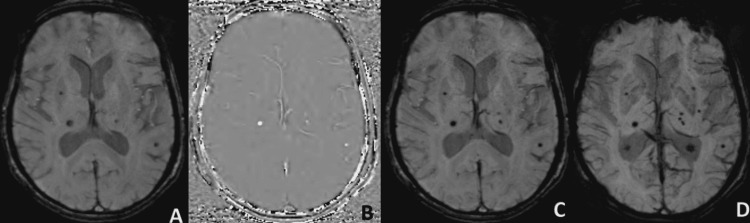
Magnitude (A), Phase (B), SWI (C), and mIP (D) images of the SWI sequence show hypointense areas on magnitude images, hyperintense on phase in the right gangliocapsular region in a known case of chronic hypertension suggestive of microhemorrhage due to hypertensive angiopathy.

Extravasated hemoglobin turns into deoxyhemoglobin, a paramagnetic material that produces inhomogeneity. SWI can identify very minute hemorrhages within the infarct because it is extremely sensitive to magnetic field inhomogeneity. Our study found five patients each with amyloid angiopathy (Figures 5A-5D) and hypertensive microbleed (Figures 6A-6D). Our findings corroborated the findings of Mittal et al. [4] showing increased sensitivity of SWI to identify numerous microbleeds that would otherwise go undetected on routine imaging.

Because of its vague clinical presentation, CVST is often difficult to diagnose. Direct signs of thrombosis (triangle sign and empty delta) on CT and normal flow void loss on MRI, might be undetected in cases with low clinical suspicion. Indirect indications of dural venous thrombosis include cerebral edema, infarction, and hemorrhage. If CVST is left undetected and thrombolytic treatment is not initiated promptly, it can be fatal [28]. By exhibiting venous stasis and collateral sluggish flow, SWI has become a helpful tool for assessing CVST. In the veins affected by dural sinus thrombosis, the concentration of deoxyhemoglobin rises. On SWI, this shows up as a significant hypointense signal intensity. SWI can detect hemorrhage within venous infarct at an early stage [4].

In our study, among the 10 patients with venous thrombosis, seven (70%) had hemorrhagic infarcts, followed by cortical venous thrombosis and superior sagittal sinus thrombosis. Our results are consistent with those of a study of 39 patients with cerebral venous thrombi by Idbaih et al. [29], which showed that the sensitivity of SWI for detecting clots in sinuses was 96% and 71% between day 1 and day 3.

In our study, the correlation of CT with SWI detection of CVST, microhemorrhage, and cerebral amyloid angiopathy showed better results with SWI compared to CT with statistical significance (P<0.05). Our findings were corroborative with those of Mittal et al. [4].

There were few limitations in our study such as small sample size and single-center study. The technique cannot be generalized in resource limited settings such as India, and the post-operative presence of MR incompatible orthopedic hardware and patients having a history of cardiac pacemakers, metallic foreign bodies, and cochlear implants in-situ were excluded from the study. Also, use of SWI in assessing the patients post thrombolytic therapy was not done. Further research that is multi-centered and with a large sample needs to be carried out.

## Conclusions

SWI is a useful imaging sequence that provides relevant information in stroke patients and requires only an additional three to four minutes to perform. SWI can identify various features such as hemorrhage, intra-arterial thrombus, or concomitant microbleed. The detection of microhemorrhage which is better seen on SWI is of prognostic value and affects therapeutic decisions and the use of thrombolytics. In our study, detection of CVST, microhemorrhage, and cerebral amyloid angiopathy showed better results with SWI when compared to CT. In conclusion, SWI is a simple, reliable, non-invasive imaging technique. SWI does not need the use of a contrast medium and can be added as an auxiliary sequence in routine stroke protocol without much increase in the scan time.
